# Advances in biomaterials for osteonecrosis treatment

**DOI:** 10.3389/fphar.2025.1559810

**Published:** 2025-05-21

**Authors:** Dapeng Wang, Jiannan Li, Yu Liu, Shuaishuai Wang, Shuo Duan, Zhiyang Liu, Shuaiwei Li, Jun Liang, Guangwei Meng, Minglei Zhang

**Affiliations:** ^1^ School of Mechanical and Aerospace Engineering of Jilin University, Changchun, China; ^2^ Department of Wound Repair, Plastic and Reconstructive Microsurgery, China-Japan Union Hospital of Jilin University, Changchun, China; ^3^ Department of Critical Care Medicine, China-Japan Union Hospital of Jilin University, Changchun, China; ^4^ Department of Orthopedics, China-Japan Union Hospital of Jilin University, Changchun, China; ^5^ Outpatient and Emergency Units, China-Japan Union Hospital of Jilin University, Changchun, China

**Keywords:** osteonecrosis, biomaterials, osteonecrosis therapy, biopolymers, polymer

## Abstract

Osteonecrosis, or ischemic osteonecrosis, occurs when bone tissue dies due to a reduced blood supply. This process begins with the death of osteocytes and is followed by the development of necrotic bone tissue. The body initiates intrinsic repair mechanisms to counteract osteonecrosis. However, insufficient blood supply and poor osteogenic microenvironments often lead to suboptimal outcomes Treatment of osteonecrosis is focused on controlling symptoms, especially pain, and preserving the function of the affected bone. In severe cases, joint replacement may be required. For early-stage patients, the main goal is to restore blood flow and encourage bone regeneration to slow or prevent further damage. While traditional treatments such as drugs and surgery are still common, there is growing interest in using biomaterials to aid bone healing and possibly avoid the need for joint replacement. This article reviews the latest progress of biomaterials for the treatment of osteonecrosis. These materials support bone repair by improving the local environment around bone, influencing cellular behavior, and even promoting gene expression. It also discusses the challenges of transferring these materials from research to clinical practice and examines emerging trends in biomaterials research. For these promising therapies to be more effective in improving outcomes for patients with osteonecrosis, a collaborative multidisciplinary approach will be essential.

## 1 Introduction

Osteonecrosis, which is also referred to as ischemic bone necrosis or aseptic bone necrosis, is the necrosis of living bone tissue in the human skeleton. The etiology of osteonecrosis is complex and multifaceted, but it is consistently associated with the interruption of blood supply, whether due to traumatic factors such as injury and non-traumatic factors such as drinking alcohol, using corticosteroid or autoimmune diseases (Assouline-Dayan et al., 2002). The pathological progression of osteonecrosis begins with osteocyte death caused by insufficient blood flow, resulting in the development of dead bone tissue. Over time, revascularization begins as the necrotic bone re-establishes connections with reactive tissue. Newly formed blood vessels facilitate the resorption of necrotic bone through osteoclastic activity, while osteoblasts simultaneously synthesize new bone in an attempt to restore the affected area. However, this reparative process often lacks regulation, and the orientation of new bone formation may not effectively bridge the bone defect. Consequently, the structural stability of the bone deteriorates, ultimately failing to maintain sufficient mechanical strength. This disruption leads to stress fractures, bone collapse, and cartilage degradation, culminating in extensive areas of necrosis (Mankin, 1992).

Clinical symptoms of osteonecrosis exhibit considerable variability among individuals and across different affected sites; however, they typically adhere to a developmental pattern characterized by phases of “asymptomatic-painful-dysfunctional.” This progression renders the treatment of osteonecrosis predominantly symptom-driven, with the functional status of the bone acting as a pivotal determinant within the treatment framework. In instances where a patient has lost normal bone function, the treatment plan generally necessitates prosthetic replacement surgery, a procedure commonly indicated for cases of joint osteonecrosis (Issa et al., 2013; Seyler et al., 2007). Conversely, when the patient’s bone maintains normal function, the primary treatment objectives focus on alleviating pain, delaying the progression of necrosis, and preserving bone function. In this context, the central tenet of treatment strategy is to restore or enhance blood supply to the compromised bone tissue. Common therapeutic approaches encompass conservative management strategies, including pharmacological treatment and physiotherapy (Mont et al., 2006), as well as surgical interventions such as decompression and bone grafting (Sultan and Mont, 2019; Herrera-Soto and Price, 2011).

With a deeper understanding of osteonecrosis treatment, the focus has shifted towards controlling its progression and avoiding prosthetic replacement, which places high demands on effective bone regeneration. Advancements in biomaterials science have revealed numerous materials that promote bone regeneration, underscoring their significant potential for treating osteonecrosis. These biomaterials facilitate bone regeneration through mechanisms such as modifying the microenvironment, regulating cellular activities, promoting gene expression, and other related processes. This review offers an in-depth summary of the biomaterials being explored for the treatment of osteonecrosis, discussing their mechanisms of action, clinical applications, and emerging trends in biomaterials technology. By analyzing recent studies, this article highlights the importance of advanced materials in promoting bone repair and achieving structural restoration. Additionally, we address the challenges of clinical translation and outline future research directions, emphasizing the need for multidisciplinary approaches to optimize these innovative solutions for improved patient outcomes in managing osteonecrosis.

## 2 Bioceramics

Bioceramics, in its broadest sense, are all ceramics that have the potential to serve as biomaterials. This review categorizes bioceramics into five groups based on their crystal structure and Ca/P ratio: apatitic and non-apatitic calcium phosphates, magnesium-based, silicate-based, and trace element-doped ceramics (Rajendran et al., 2024). When implanted into the body, these materials exhibit bioactive properties that allow them to interact and integrate with bone tissue. Bioceramics are particularly beneficial in medical treatments, such as the management of osteonecrosis, due to the promotion of bone and blood vessel regeneration via this interaction. Detailed descriptions of the mechanisms by which each type of bioceramic promotes osteogenesis are provided below:

### 2.1 Apatitic calcium phosphates

Apatitic calcium phosphates are inorganic compounds with a high Ca/P ratio, typically ranging from 1.5 to 1.67. This ratio is strikingly similar to human bone, enhancing the materials with superior mechanical strength and compatibility. Due to these qualities, apatitic calcium phosphates are extensively used in the treatment of orthopedic conditions. Among them, hydroxyapatite (HA) stands out as the most prominent example. The following section will examine HA as a case study to illustrate the osteogenic mechanisms of these materials.

HA materials can promote osteogenesis. After implantation, HA can release Ca^2+^ and PO_4_^3−^ ions, which re-precipitate on its surface, forming a hydroxyapatite layer that resembles natural bone. The hydroxyapatite layer can promote bone cell adhesion, growth, and differentiation on its surface, while also adsorbing osteogenic proteins and growth factors from the bloodstream. The sustained release of Ca^2+^ and PO_4_^3−^ ions creates a microenvironment around the implant site that is highly supportive of bone regeneration. HA’s surface roughness and porous structure enhance cell-material interactions, providing an ideal three-dimensional environment for osteocyte adhesion and growth. As a result, the formation of new vessels within the bone supports the healing process by promoting tissue repair (Samavedi et al., 2013).

In addition to its inherent osteogenic properties, HA can also form osteogenic scaffolds with other materials. HA can form composite scaffolds for two main reasons. Firstly, the chemical structure of HA can be modified to create different forms that match the binding requirements of various materials (Karakeçili et al., 2022; Haider et al., 2017). Furthermore, HA demonstrates excellent biocompatibility and biodegradability, allowing it to serve as a temporary framework that is progressively replaced by bone tissue as osteogenesis advances (Hou et al., 2022; Turon et al., 2017). Synthetic polymers like poly(lactic-co-glycolic acid) (PLGA), natural polymers like silk fibroin, chitosan (CS), and alginate (ALG) (Bhattacharjee et al., 2017; Haider et al., 2024; Gholap et al., 2024; Damiri et al., 2024), and a variety of components like HA can be mixed with these to form scaffolds (Barcena et al., 2024; Cavelier and Hutmacher, 2024; Jia et al., 2024; Khan et al., 2023; Barcena et al., 2024; Cavelier and Hutmacher, 2024; Jia et al., 2024; Khan et al., 2023).

**FIGURE 1:**
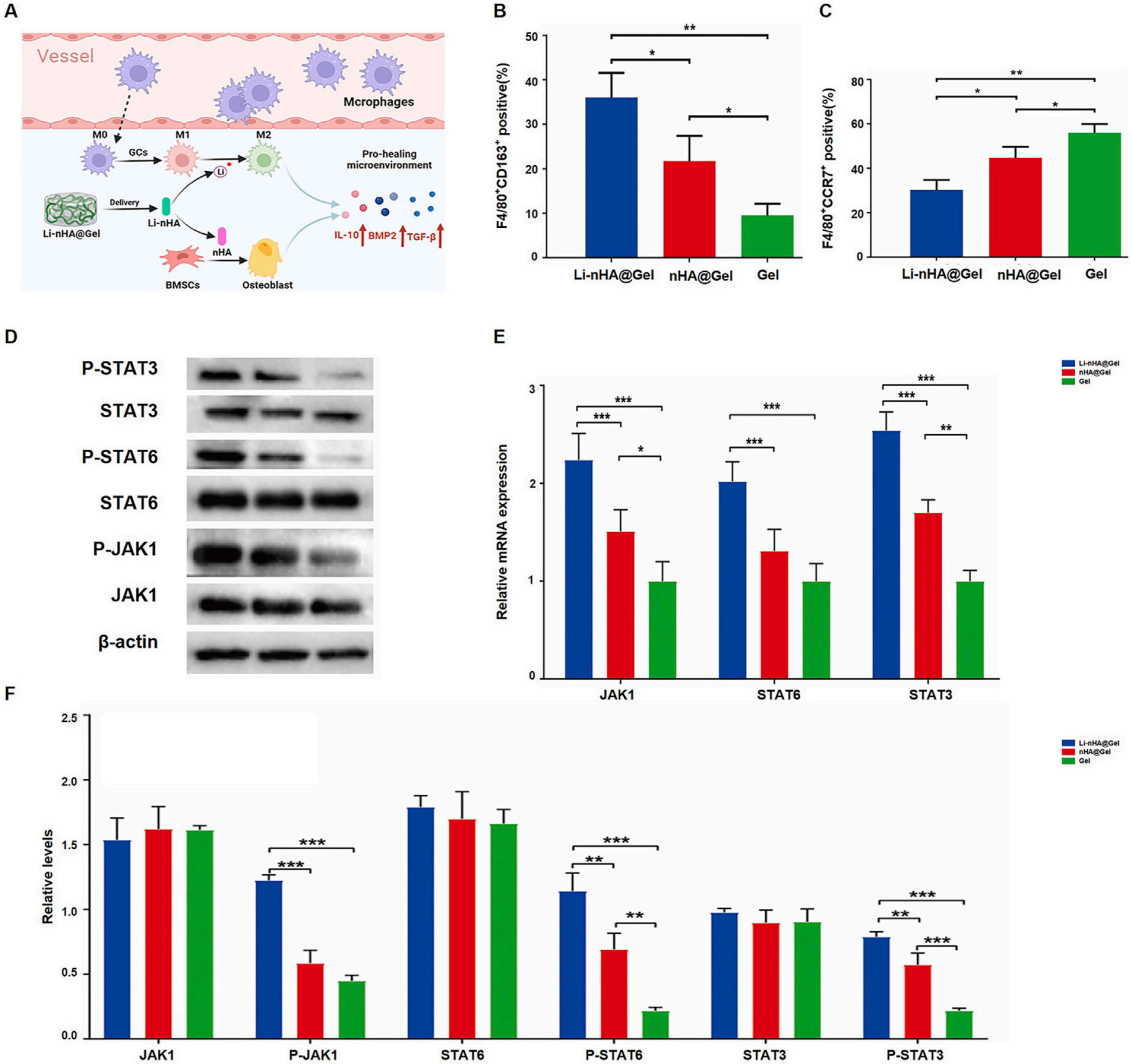
Li-nHA@Gel promotes bone repair via JAK1/STAT6/STAT3 activation: **(A)** Enhances macrophage polarization; **(B)** Increases M2 macrophages; **(C)** Reduces M1 macrophages; **(D–F)** Boosts STAT3/STAT6 phosphorylation and mRNA expression (*p < 0.05, **p < 0.01, ***p < 0.001, which indicates significant differences in osteogenic marker expression between Li-nHA@Gel and control groups, as determined by ANOVA.). [Reproduced with permission (22), Copyright 2024, Elsevier].

Incorporating other materials into HA scaffolds enhances their toughness and mechanical strength, while maintaining the material’s superior osteogenic potential. It provides a stable platform for the delivery of metals, osteogenic drugs, and bioactive factors, making the osteogenic composite materials formed with scaffolds highly promising for the treatment of osteonecrosis. Various studies have built upon this idea by exploring composite materials designed to enhance bone healing and tissue regeneration. Luo et al. (Cheng et al., 2023) developed a lithium, nano hydroxyapatite, and hydrogel (Li-nHA@Gel) composite designed to promote bone tissue repair and regeneration by releasing lithium ions, which activate the JAK1/STAT6/STAT3 signaling pathway (Figure 1A). In the Li-nHA@Gel treatment group, the percentage of F4/80+CD163+ cells significantly increased, while the percentage of F4/80+CCR7+ cells decreased, suggesting that the material facilitated M2 macrophage polarization while suppressing M1 macrophage polarization (Figures 1B, C). Furthermore, the expression and phosphorylation levels of proteins involved in the JAK1/STAT6/STAT3 signaling pathway were notably elevated in the treatment group (Figure 1D). The quantitative analysis of the relative mRNA expression levels of JAK1, STAT6, and STAT3, as well as the relative protein expression levels of JAK1, P-JAK1, STAT6, P-STAT6, STAT3, and P-STAT3, across various treatment groups, further supported these findings. This additional data strengthens the evidence that the Li-nHA@Gel composite effectively modulates the signaling pathways crucial for bone repair and regeneration (Figures 1E, F). Cheng et al. (2023) created a nanohydroxyapatite/CS@polydopamine-strontium composite (nHA/CS@PDA-Sr) to promote bone repair. Similarly focusing on Sr Zhuang et al. (2023) demonstrated that Sr-HA bioceramics, which contain strontium, can promote osteogenesis via stimulation of the Erk1/2 MAPK and PI3K/AKT pathways. Li et al. (2023a) formulated a multifunctional hydrogel scaffold incorporating polyvinyl alcohol, gelatin (GL), sodium alginate, aspirin, and nano hydroxyapatite. This research validated the combined effects of aspirin and nano-hydroxyapatite in promoting osteogenesis and exhibiting anti-inflammatory activity. Zeng et al. (2023) engineered hollow hydroxyapatite microspheres (HHMs) were integrated with chitosan (CS) to formulate a composite scaffold infused with recombinant human C-X-C motif chemokine ligand 13 (rhCXCL13-HHM/CS). The findings indicated that, 12 weeks post-implantation, the rhCXCL13-HHM/CS scaffold markedly enhanced bone regeneration and vascular remodeling. The osteogenic effect of the rhCXCL13-HHM/CS scaffold was facilitated by the PI3K-AKT signalling pathway. Firouzeh and colleagues (Firouzeh et al., 2024). Engineered a decellularized amniotic membrane (DAM) scaffold impregnated with hydroxyapatite (DAM-HA). The findings demonstrated that the DAM scaffold successfully promoted stem cell viability and expansion, with the addition of hydroxyapatite greatly enhancing osteogenic differentiation. In conclusion, combining hydroxyapatite with various substances to form composite materials improves its mechanical strength while supporting osteogenesis and promoting tissue regeneration. These developments highlight the potential of composite biomaterials in addressing osteonecrosis and other bone-related conditions.

### 2.2 Non-apatitic calcium phosphates

Non-apatitic calcium phosphates have a lower calcium/phosphorus ratio than apatitic calcium phosphates. Calcium phosphates that are not apatitic include amorphous tricalcium phosphate (ACP), dicalcium phosphate dihydrate (DCPD), dibasic calcium phosphate (DCP), octacalcium phosphate (OCP), and α, β-tricalcium phosphate (TCP) (Laskus and Kolmas, 2017).

The osteogenic potential of non-apatitic calcium phosphates is driven by two main characteristics. Firstly, their high solubility and absorbability enable them to dissolve efficiently in the body, releasing calcium and phosphate ions vital for bone mineralization, which makes them effective for bone repair (Lowe et al., 2019). Their moldability enables the creation of customized implants for complex bone defects, while their injectability allows for minimally invasive delivery to hard-to-reach areas. Secondly, their ion-exchange capacity can be enhanced by doping with trace elements such as Mg^2+^, Zn^2+^, Si^4+^. These ions help stabilize the phosphate structure while enhancing bone regeneration and vascular remodeling, thus accelerating bone and blood vessel formation (Bandyopadhyay et al., 2006). By leveraging these properties, non-apatitic calcium phosphates can be optimized for specific therapeutic outcomes, making them highly versatile in orthopedic applications and bone tissue engineering.

Given these properties, non-apatitic calcium phosphates are frequently combined with various materials to form composites that promote osteogenesis. Yuan et al. (2024) by combining γ-TCP with varying percentages of magnesium silicate (MS, Mg_2_SiO_4_), ranging from 10% to 30%, composite bioceramic scaffolds were produced using 3DF technology. Stem cells from mouse bone marrow (mBMSCs) and human umbilical vein endothelial cells (HUVECs) were able to adhere, proliferate, and remain viable with the help of these scaffolds. They also enhanced alkaline phosphatase function and increased gene expression in pathways related to angiogenesis and osteogenesis, utilizing 3D printing technology. Niu et al. (Obata et al., 2017) successfully developed PCL/Ŏ-TCP composites for personalized repair of extensive bone lesions. The composite’s osteogenic potential was maximized when the β-TCP content reached 20%, the research found that MC3T3-E1 cells, which are a kind of mouse pre-osteoblastic cell line, were significantly encouraged to multiply and attach to the composite material. According to He et al. (2024) studied the impact of doping with Si, Zn, and a combination of the two on the angiogenic and osteogenic activities of γ-TCP in a laboratory setting. It was found that Si-TCP, Zn-TCP, and Si/Zn-TCP were all very biocompatible. More precisely, the angiogenic potential of ο-TCP was greatly improved by Si-TCP, and its osteogenic properties were markedly improved by Zn-TCP. Notably, Si/Zn-TCP exhibited exceptional bifunctionality, promoting both new blood vessel formation and bone growth. Taken together, these findings suggest that composites of non-apatitic calcium phosphate can enhance both osteogenesis and angiogenesis, which makes them good options for future uses in advanced bone tissue engineering.

### 2.3 Silicate-based ceramics

The most representative compound of silicate-based ceramics is bioglass, primarily composed of Si, Ca. Bioactive glass not only promotes osteogenesis but also provides mechanical support and bone integration, similar to other bioceramics. Its unique advantage lies in its sustained ion release, which further enhances bone formation. Firstly, the basic ionic components of bioglass, Si and Ca can contribute to osteogenesis (Obata et al., 2017; Varanasi et al., 2009). Additionally, bioglass can be loaded with osteogenic ions like Mg^2+^, Cu^2+^, Zn^2+^, and Sr^2+^ (Kargozar et al., 2022; Shendage et al., 2024; Weng et al., 2017; Saino et al., 2011; Fredholm et al., 2012). The ions are released gradually owing to the unique structure of bioglass, forming a conductive layer on the bone surface that supports an ideal microenvironment for bone healing.

Since Hench et al. (1971) discovered the first silicate-based bioglass, known as 45S5 bioactive glass, a SiO_2_–CaO–P_2_O_5_based biomaterial, this invention has laid the foundation for the development of bioglass. In recent years, research on bioactive glass has increasingly focused on the development of composite materials by combining bioactive glass with other materials. Xiong et al. (2023) created a collagen- and naringin-loaded mesoporous bioglass/poly(L-lactic acid) composite scaffold (NG-MBG/PLLA) to aid in bone regeneration. The results demonstrated that this scaffold improved mBMSC proliferation and differentiation. It also reduced reactive oxygen species (ROS) production in response to lipopolysaccharide stimulation and increased calcium nodule formation and alkaline phosphatase activity in mBMSCs under macrophage-conditioned environments. By Dai et al. (2024) For osteogenesis research, a 3D-printed scaffold (PLGA/PCL/MgMNBG) was created by combining magnesium-containing micro-nano bioactive glass with PLGA and PCL. The results indicated that the PLGA/PCL/MgMNBG scaffold had the ability to modify macrophages in order to suppress inflammatory responses, thereby promoting angiogenesis and osteogenesis. Kido et al. (2023) proposed a bone regeneration strategy using a composite material made of bone marrow stromal cells and a bioactive glass/collagen scaffold. The results indicated that this composite material efficiently promoted bone formation and notably enhanced the expression of markers related to bone repair. Jeyachandran et al. (2023) developed a Bioglass-poly(lactic-co-glycolic acid) and fibrin composite construct to support endochondral bone formation (Bg-PLGA@fibrin) designed to enhance endochondral ossification. The research demonstrated that the composite effectively stimulated mesenchymal stem cell hypertrophy, matrix mineralization, and osteogenic differentiation. It notably facilitated various stages of endochondral ossification via sequential material signalling, obviating the necessity for external inducing factors. This method possesses considerable potential for clinical application by diminishing both the pre-implantation *in vitro* culture duration and the intricacy of employing external inducers. Janmohammadi et al. (2024) Created a 3D-printed composite scaffold by integrating alkaline-treated PCL with astragalus gum and 45S5 bioglass (M-PCL/TG-BG) for the restoration of bone defects. The findings showed scaffold markedly facilitated bone regeneration in rat calvarial defects, improving bone mineral density (BMD) and the bone volume/total volume ratio (BV/TV). Histological and gene expression analyses confirmed that the scaffold enhanced bone integration and repair by upregulating osteogenic genes, including Runx2 and type I collagen. These results emphasize the significant potential of bioactive glass composites in enhancing bone regeneration, osteogenesis, and tissue integration, presenting promising opportunities for advanced therapeutic strategies in bone defect repair and clinical applications.

### 2.4 Magnesium-based ceramics

During osteogenesis, Mg^2+^ is involved in a number of different processes. Osteogenesis is supported by its ability to promote angiogenesis and anti-inflammatory effects, as well as by its role in regulating the balance between bone resorption and formation and in enhancing mineralization and osteoblast proliferation and differentiation (Gu et al., 2019; Cabrejos-Azama et al., 2014; Kaiser et al., 2022; Hu et al., 2023; Samanta et al., 2019; Liu et al., 2021). Scaffolds and bone cement are the two most common applications of this ceramic type for the purpose of fostering osteogenesis.

A common type of magnesium-based ceramic is composite phosphate-based scaffolds. To encourage cell attachment, growth, and the formation of new bone tissue, these scaffolds generally possess a porous design that resembles the natural three-dimensional structure of bone. They provide mechanical support while consistently releasing Mg2+, thereby augmenting the osteogenic process. Gu et al. (2019) fabricated Mg2+-doped beta-tricalcium phosphate scaffolds through cryogenic three-dimensional printing and subsequent sintering. The scaffolds exhibited an interlinked porous architecture and compressive strength akin to cancellous bone. Mg^2+^ significantly enhanced the proliferation, viability, and expression of genes related to osteogenesis and angiogenesis in human bone marrow stem cells (hBMSC) and HUVEC, underscoring their potential for enhanced bone regeneration and angiogenesis. Wei et al. (2010) engineered 3D microporous/macroporous Mg2+-CaSO4 (micro/ma-MCP) scaffolds exhibiting 52%–78% porosity through a leaching technique. These scaffolds enhanced MG-63 cell attachment, growth, and alkaline phosphatase (ALP) activity, while also enhancing degradation in Tris-HCl solution. *In vivo* studies demonstrated superior biocompatibility, biodegradability, and expedited bone regeneration. Adhikari et al. (2016) Engineered a 3D scaffold utilizing chitosan (CS), carboxymethyl chitosan (CMC), calcium phosphate monobasic, and magnesium oxide (MgO) in tissue engineering. The scaffold, distinguished by highly interconnected pores (100–300 µm), emulates the architecture of natural bone and liberates Mg2+ and Ca2+ ions, thereby enhancing osteoblast activity and biomineralization, rendering it appropriate for bone regeneration applications. These studies collectively illustrate the capacity of Mg2+-based composite scaffolds, characterized by their porous structures and ion-releasing capabilities, to substantially improve osteogenesis and bone regeneration in tissue engineering applications.

**FIGURE 2:**
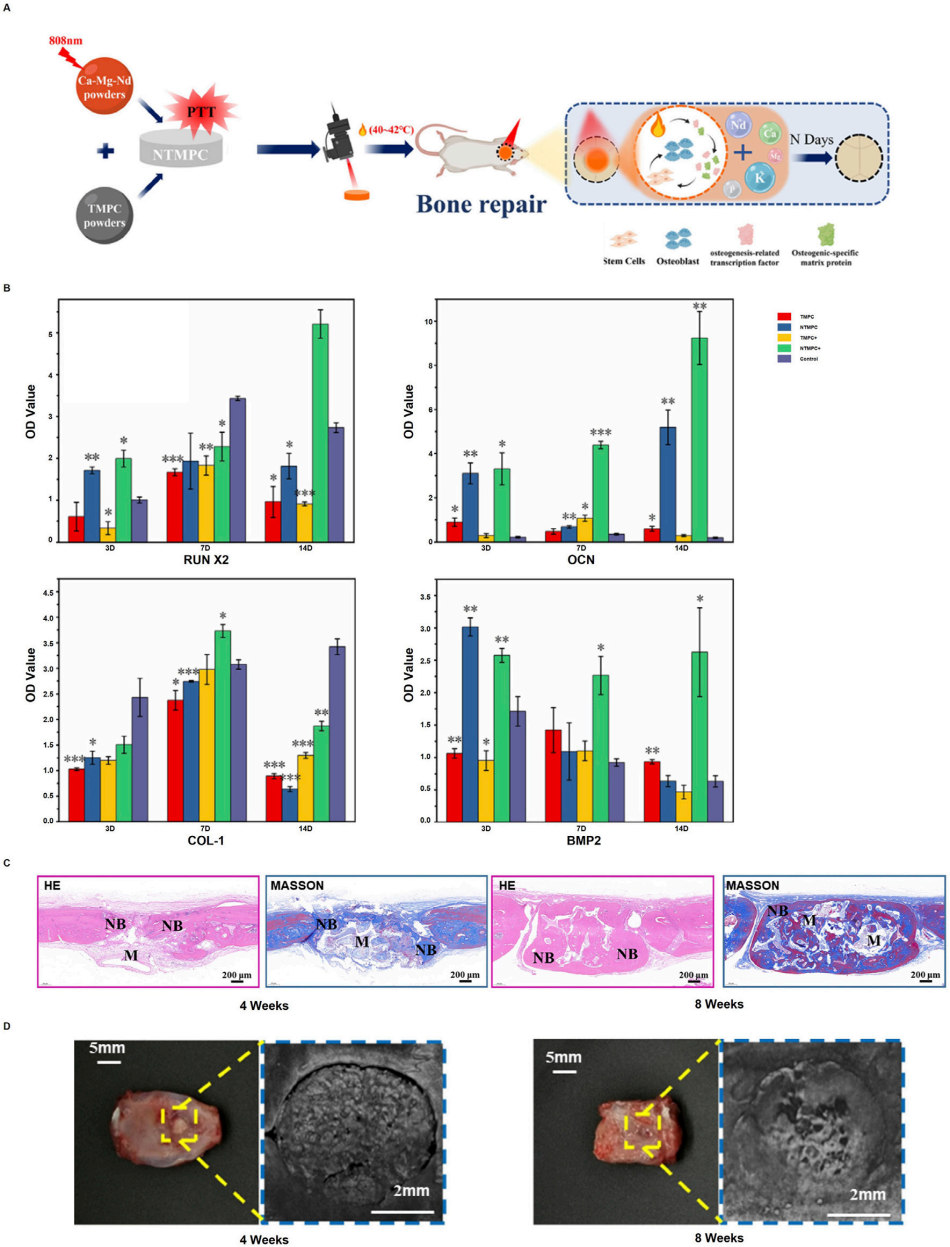
NTMPC promotes bone repair: **(A)** Conditions (40°C–42°C, 5 min, 2D/time); **(B)** Osteogenic markers (RUNX2, OCN, COL I, BMP-2) with NTMPC and NIR; **(C)** H&E and Masson staining; **(D)** Pro-osteogenic effect at 4W and 8W. (*p < 0.05, **p < 0.01, ***p < 0.001 vs. β-TCP). [Reproduced with permission (55), Copyright 2024, Elsevier].

Bone cement, once hardened, can exhibit biocompatibility and degradability similar to bioceramics. Using magnesium-based ceramics in the form of bone cement for osteogenesis not only improves the bone microenvironment but also helps to match the rate of bone formation. Hou et al. (2024) developed a bone cement rich in Mg^2+^, Ca^2+,^ and Nd^3+^ (NTMPC), composed of trimagnesium phosphate cement (TMPC) and Ca-Mg-Nd powder. When heated to 40°C–42°C using an 808 nm laser, NTMPC can modulate osteogenic cells and biofactors to promote bone repair (Figure 2A). The NTMPC group co-cultured with bone marrow stem cells (BMSCs), which composed NTMPC+, showed significantly higher gene and protein expression levels of RUNX2, OCN, COL-1, and BMP2 compared to the control groups (Figure 2B), H&E and Masson’s staining, along with pro-osteogenic markers, were used to assess effect revealed that the NTMPC+ group formed more new bone and mineralized tissue at 4 and 8 weeks (Figures 2C, D). Eugen et al. (2023) studied the biosorption of 3D-printed magnesium phosphate (MP) ceramics using human osteoclast cultures. While MP ceramics showed significant chemical dissolution, no osteoclast-mediated resorption occurred. The biocompatible and bioactive MP ceramics are expected to enhance bone regeneration and fully degrade within 1.5–3.1 years, offering a faster alternative to calcium phosphate grafts. Cabrejos-Azama et al. (2014) synthesized magnesium-doped calcium phosphate cements (Mg-CPC) and found they enhanced osteoblast proliferation and bone regeneration compared to undoped CPC. Mg-CPCs showed improved cytocompatibility and bone formation, indicating their potential for personalized bone therapy. Lukina et al. (2023) developed magnesium calcium phosphate ceramics for bone restoration, featuring Mg^2+^ and Ca^2+^ for controlled resorption. The ceramics maintained their shape during degradation, and setting inhibitors optimized their composition. *In vivo* studies confirmed the material’s potential for bone restoration, with magnesium ions influencing resorption rates. Kaiser et al. (2022) developed magnesium phosphate cements (MPCs) for osteogenesis by reducing the powder-liquid ratio to increase the content of highly soluble phases such as struvite (MgNH_4_PO_4_.6H_2_O) and K-struvite (MgKPO_4_.6H_2_O). The study demonstrated that these composite materials enhance osteogenesis by speeding up degradation, facilitating quick bone ingrowth and concurrent new bone formation as the cement breaks down. To conclude magnesium-based ceramics and cements hold great promise for improving bone regeneration by enhancing osteogenic environments and achieving optimal degradation rates.

### 2.5 Trace element-doped ceramics

`The concept of trace element-doped ceramics was first summarized by Rajendran et al. in their review (Rajendran et al., 2024). They proposed that certain trace elements naturally exist in the structure of bone, and doping these elements into bioceramics further enhances their osteogenic and angiogenic capabilities. These trace elements include Mg, Mn, Li, Sr, Cu, Ce, Eu, La, and Gd. Although ceramics doped with various trace elements have been applied, Li and Cu are the most frequently studied. Below is a detailed explanation of the applications of Li- and Cu-based ceramics.

Li^+^ supports osteogenesis by modulating the expression of genes associated with bone formation and boosting cell activity (Alicka et al., 2019). Qiu et al. (2021) improved the osteogenic potential of PEEK implants by applying a nanocomposite coating of albumin and Li^+^-bioactive glass nanospheres using dip-coating. The coating made PEEK more hydrophilic and rough, thereby enhancing the attachment, growth, and osteogenic differentiation of bone mesenchymal stem cells. Such findings bode well for improved osteointegration. Tseng et al. (2023) developed diopside-Li_2_O bioceramics with improved strength, biodegradation resistance, and bioactivity. Li0.25 (25 mol% Li_2_O) showed optimal hardness, minimal weight loss, stable pH, and good cell viability, making it promising for bone implants. Li et al. (2024) improved lithium disilicate (LD) glass-ceramics for bone regeneration through Li+/Na+ ion exchange. This process promoted hydroxyapatite formation and boosted cell attachment, growth, and osteogenic differentiation. The findings suggest Li+/Na+ exchange as a promising method to enhance LD glass-ceramics for orthopedic use. These studies collectively demonstrate the potential of Li-based ceramics in orthopedic applications, showing that Li+ can significantly enhance bioactivity, mechanical strength, and osteointegration. This renders them as promising candidates for bone regeneration and repair.

Cu^2+^ promotes osteogenesis by regulating the immune response, increasing the expression of bone-related genes and proteins, and supporting the growth and differentiation of osteoblasts (Huang et al., 2019; Huang et al., 2018; Zheng et al., 2018). Huang et al. (2019) examined a Cu-containing micro/nano-topographical surface (Cu-Hier-Ti) and discovered that Cu^2+^ release stimulated M1 macrophages, consequently facilitating osteogenesis and improving bacterial defence. *In vivo*, Cu-Hier-Ti improved osteointegration and osteogenic markers, indicating potential for bone regeneration. Huang et al. (2018) used micro-arc oxidation to create copper-infused ceramic coatings on titanium implants. These Cu coatings promoted M1 macrophage polarization, enhancing osteogenesis and antibacterial activity. The inclusion of Cu in biomaterials demonstrates potential for improving osteogenic and antimicrobial properties. Wu et al. (2023a) improved PEEK through the functionalization of its surface with Cu-Sr bilayer bioactive glass nanoparticles (CS-BGNs) utilizing polydopamine (PDA). The regulated release of Cu2+ and Sr2+ influenced macrophage polarization, facilitating initial antibacterial responses and subsequent osseointegration. This method enhances the osteogenic and antibacterial characteristics of PEEK, presenting a novel strategy for immunomodulatory biomaterials. The integration of copper into biomaterials presents a viable approach to augmenting osteogenic and antimicrobial characteristics, facilitating the development of advanced bone regeneration technologies.

## 3 Natural biopolymers

Natural biopolymers are a class of polymeric biomaterials derived from biological sources. Some natural polymers not only exhibit excellent biocompatibility and low immunogenicity but also possess osteogenic properties. The main types of osteogenic natural polymers include gelatin, chitosan and alginate. Below is a detailed description of how these three natural biopolymers promote osteogenesis.

### 3.1 Gelatin

GL is a natural biopolymer derived from the hydrolysis of collagen found in animal bones, tendons, and skin using acidic or alkaline methods. The abundant RGD sequences in GL promote cell adhesion by interacting with integrins, making it beneficial for tissue regeneration. Additionally, GL exhibits low antigenicity and offers excellent potential for chemical modification. What’s more, it readily integrates with a variety of natural and synthetic polymers, making it ideal for creating osteogenic scaffolds (Ranganathan et al., 2019).

Zhou et al. (2022) developed an injectable dual-crosslinked hydrogel made of GL, alginate dialdehyde, calcium ions, and borax for osteonecrosis repair. The hydrogel enhanced mechanical properties, conforming to complex facial bone defects and withstanding masticatory forces. With added nHA, it formed a bioactive porous structure that promoted interactions between macrophages and BMSCs, effectively enhancing bone regeneration and repairing critical-sized cranial defects. Xu et al. (2023) investigated the application of GL in biphasic calcium phosphate (BCP)/gelatin methacrylate (GLMA) composite hydrogels. Their study showed that the composite hydrogel demonstrated excellent biocompatibility and promoted the osteogenic differentiation of bone marrow mesenchymal stem cells(BMMSCs), leading to significant new bone formation in rat cranial defects. A hydrogel composed of GL (GL-HAlg-DN) was developed by Wu et al. (2023b) to imitate the extracellular matrix and repair osteonecrosis. Hydrogels are biodegradable and have stable swelling in addition to improved mechanical properties (0.9 MPa tensile strength, 177% elongation). Sinapene-loaded GL-HAlg-DN hydrogel, a promising scaffold for tissue engineering, significantly improved bone regeneration in a rat bone lesion model. To address the repair of alveolar bone defects, Wang et al. (2024a) created a thermosensitive/photosensitive GLMA gel by means of a freeze-ultraviolet (FUV) technique. Through the p38 MAPK pathway, the gel encouraged mandible rebuilding, bone formation and blood vessel development, both in laboratory settings and in animal models. And it was highly biocompatible and easy to manufacture.

Electrospinning serves as a crucial method for utilizing GL in the field of bone regeneration. By generating nanofiber scaffolds that replicate the extracellular matrix (ECM), it offers an expanded surface area to support cell attachment and proliferation, facilitating tissue repair. When combined with other materials in composite scaffolds, the proportion of GL affects the fiber diameter and morphology, thereby affecting both the mechanical and biological characteristics of the scaffold. Optimizing fiber surface morphology, porosity, and bead-free structure enhances cell adhesion, proliferation, and differentiation, creating an ideal microenvironment for bone tissue regeneration and improving repair outcomes (Ranganathan et al., 2019). Pankongadisak et al. (2020) developed electrospun GL mats containing plasmid DNA (pDNA) polyplexes for regenerative medicine. The pDNA, condensed with lipid-modified polyethyleneimine (PEI) and poly(aspartic acid) (pAsp), was electrospun into fibers (150–350 nm) with a particle size of 82 nm and a +20 mV zeta potential. Polyethylene glycol improved pDNA entrapment (∼71%) and transfection efficiency. pDNA encoding BMP-2 significantly induced ALP activity, promoting osteogenic differentiation. These GL mats hold promise for tissue regeneration. Ola et al. (2024) enhanced the bone regeneration potential of electrospun GL scaffolds by incorporating diatomite earth (DE) biosilica at 1%, 3%, and 5% loadings. DE made the scaffold more rigid and less swollen, and FG-DE3 had the greatest preosteoblast response and mineralization. Nevertheless, cell activity was negatively affected by the 5% DE loading. There is hope for bone tissue engineering with these DE-loaded scaffolds, particularly FG-DE3. In their study, Bochicchio et al. (2020) created a bone tissue engineering electrospun scaffold out of poly(d,l-lactide), GL, and RKKP glass-ceramics. The biomineralization process, cell survival, and osteogenic differentiation were all increased by RKKP, which contains La^3+^ and Ta^5+^ ions. Incubation in a bodily fluid simulator verified the development of hydroxyapatite. The varying RKKP content directed canine stem cell differentiation toward either chondrogenic or osteogenic pathways. Cardoso et al. (2024) developed a GLMA electrospun scaffold with 5% naringenin (NA) for bone tissue engineering. The GLMA + NA5% scaffold enhanced cell proliferation, osteogenic differentiation, and mineralized nodule formation while reducing inflammation and promoting collagen synthesis. Behere et al. (2021) developed a biodegradable scaffold combining PCL, GL, and nano-hydroxyapatite (nHAp) to enhance bone formation. Cell proliferation, osteogenic differentiation, and cell survival were all enhanced by the PCL-GL-nHAp scaffold in comparison to PCL alone. Its potential as a substrate for repair was demonstrated by its considerable increase in a alkaline phosphatase (ALP) level and calcium mineralization. Electrospun GL-based composite scaffolds have shown promise in these experiments, and their capacity to improve cell survival, osteogenic differentiation. The ability to promote bone healing and regeneration makes these approaches valuable for advancing the field of bone tissue engineering.

### 3.2 Chitosan

**FIGURE 3:**
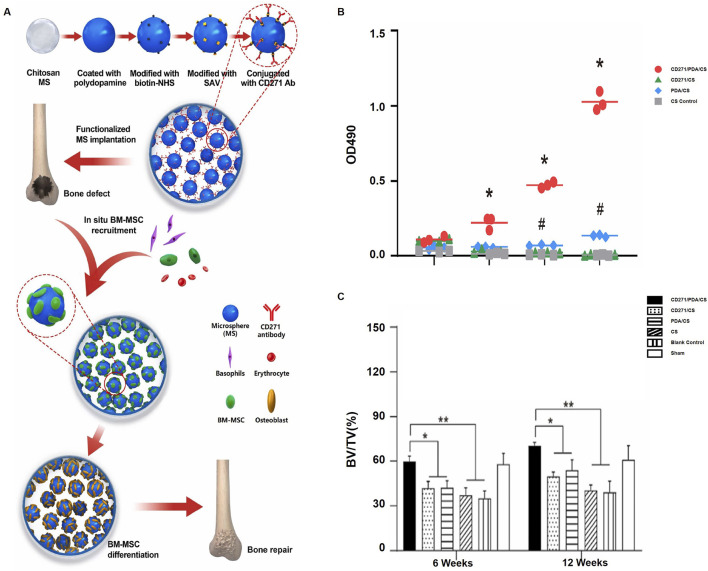
Chitosan microspheres functionalized with CD271 enhance bone regeneration by recruiting bone marrow stromal cells: **(A)** BM-MSCs are recruited *in vivo* onto chitosan microspheres functionalized with CD271; **(B)** The proliferation of BM-MSCs on various microspheres is measured using MTS (*p < 0.05 for CD271/PDA/CS vs. CD271/CS and CS, #p < 0.05 for PDA/CS vs. CD271/CS and CS); **(C)** The bone volume fraction in the femoral condyle of rats is measured at 6 and 12 weeks (*p < 0.05, **p < 0.01). [Elsevier, 2022, Copyright, Reproduction with Permission (Garske et al., 2020)].

CS is a polysaccharide derived from crustacean shells that is well-known for its ability to be biocompatible and provide support functions. Utilizing its binding affinity for cell surface receptors, CS effectively promotes the growth and differentiation of osteogenic cells. Furthermore, it can be utilized as a scaffold to aid in the regeneration of bone defects, offering support and structure that facilitates the growth of new bone tissue (Levengood and Zhang, 2014). Peyravian et al. (2024) developed an injectable OCMC-CMCS hydrogel with angiogenic peptide QK. The hydrogel, cross-linked via a Schiff base reaction, promoted cell proliferation, angiogenesis, and inhibited femoral head necrosis, showing strong therapeutic potential in both molecular and histological evaluations. The study by Jia et al. (2019) explored CS-based hydrogels for joint cartilage repair. Encasing the synovial fluid mesenchymal stem cells (rbSF-MSCs) within the hydrogel showed enhanced compatibility and better healing results, highlighting the potential of CS in cartilage tissue engineering. CS-based hydrogels for joint cartilage repair. Encapsulating synovial fluid mesenchymal stem cells (rbSF-MSCs) in the hydrogel showed superior biocompatibility and improved repair outcomes, highlighting CS potential in cartilage tissue engineering. Sun et al.(2022) developed CD271 antibody-functionalized chitosan (CS) microspheres by coating the CS with polydopamine (PDA), modifying it with biotin-NHS, and conjugating CD271 antibodies. This approach aimed to recruit BMMSCs for *in situ* bone regeneration (Figure 3A). The CD271/PDA/CS group significantly enhanced BM-MSC recruitment compared to control groups, demonstrating the functionalized microspheres’ effectiveness in attracting stem cells (Figure 3B). Bone regeneration was also significantly improved in the CD271/PDA/CS group over 6 and 12 weeks, showcasing the scaffold’s potential in promoting bone formation and repair (Figure 3C). Sungkhaphan et al. (2023) developed a CS-based hydrogel loaded with clindamycin and geranylgeraniol for treating MRONJ-B. The hydrogel exhibited prolonged drug release, antibacterial properties, and minimized cytotoxicity induced by zoledronic acid, along with low acute toxicity both *in vitro* and *in vivo*. These findings collectively highlight the versatility and promise of CS-based hydrogels in enhancing bone and cartilage regeneration, Given their unique properties and potential uses in tissue engineering and regenerative medicine, these scaffolds show strong commercial viability.

### 3.3 Alginate

ALG, a linear polysaccharide extracted from brown algae., is recognized for its biocompatibility and ability to form gels. Alginate forms gel structures that can fill bone defects, promoting bone cell proliferation and repair. Its gelation speed and stability can be modified chemically to suit various therapeutic needs and applications (Garske et al., 2020). Guzman et al. (2021) evaluated effects of alginate composites on bone necrosis, focusing on bioactivity and drug release properties in a simulated *in vivo* environment. Their findings demonstrated that alginate composites effectively promoted bone tissue growth and repair, exhibiting good biocompatibility and controllable drug release. In their investigation into bone tissue creation, Wang et al. (2021) used a unique method that involved apg calcium peroxide (CaO_2_)/GL oxygen-releasing microspheres in conjunction with 3D-printed porous scaffolds of polycaprolactone/nano-hydroxyapatite (PCL/nHA), combined with hydrogels of ALG and GL, and BMSCs. These microspheres showed promise as a treatment for femoral head necrosis after being implanted in the core decompression area of a model. They released oxygen continuously for up to 19 days, greatly improving bone formation, angiogenesis, and cell survival. Research by Zhao et al. (2022) created an oxidized alginate that can be used to repair bone necrosis with controlled degradation kinetics. With an accelerated breakdown rate and excellent biocompatibility *in vitro* and *in vivo*, the hydrogel promoted osteogenic differentiation and BMSC proliferation. Members of the Cui group (Cui et al., 2023). Created a semi-interpenetrating network hydrogel made of ALG/GL without cross-linkers, which exhibited fast gelation (∼150 s) and high mechanical properties (compressive modulus up to 361.3 kPa). The hydrogel showed an improved biomineralization rate (Ca/P ratio ∼1.69) and a self-healing ability of 92%. This method enhanced osteoblast differentiation, proliferation, and activity. Finally, our research shows that alginate-based hydrogels have many potential applications and can enhance bone regeneration. Because of this, they are a viable choice for clinical and bone tissue engineering applications.

In the exploration of osteonecrosis treatments using natural polymers, several studies have highlighted the significant potential of combining CS and alginate in new biomaterials for this purpose. Liu et al. (2024) created an antioxidant hydrogel with selenium nanoparticles, carboxymethyl CS, and alginate (SeNPs/CMC/ALG). The hydrogel exhibited robust antioxidant activity, sustained release of selenium nanoparticles, and minimal cytotoxicity. By activating the Wnt/β-catenin pathway, this material, upon implantation following core decompression surgery, it significantly minimized femoral head necrosis while promoting bone regeneration and vessel formation. Xu et al. (2021) developed a composite implant comprising carboxymethyl CS, alginate, BMSCs, and endothelial progenitor cells (EPCs) to address steroid-induced femoral head necrosis. Angiogenesis and bone formation were both greatly improved by co-cultivating BMSC and EPC. As a potential treatment option, these implants greatly improved femoral head necrosis repair in a rabbit model by decreasing adipogenesis and increasing the survival of transplanted cells. de Almeida et al. (2018). Established the safety and efficacy of CS/sodium alginate/hydroxyapatite (Ch/NaALG/Hap) stents in the prevention of osteonecrosis. The stents successfully inhibited jaw necrosis, although they induced some inflammatory responses. An analysis of euthanized experimental rabbits confirmed the safety of the stents regarding liver and kidney function and blood parameters, thereby endorsing their clinical potential for treating jaw necrosis. These studies emphasize the promise of CS and alginate-based hydrogels in osteonecrosis treatment, offering new avenues for bone tissue engineering and clinical use.

## 4 Synthetic polymers

Synthetic polymers are man-made polymeric biomaterials, typically produced through chemical synthesis from precursor materials. Unlike natural polymers, synthetic polymers typically offer better mechanical strength and chemical durability. However, they may have limited biocompatibility and osteogenic capacity. As a result, in bone tissue engineering, synthetic polymers are frequently combined with other biomaterials or biological therapies to enhance their performance. Common synthetic polymers used in osteonecrosis treatment include PLA, PCL, and PLGA. While numerous studies have demonstrated the therapeutic benefits of synthetic polymers for osteonecrosis (ONFH), clinical applications have yet to be realized. Presented here is a summary of how these three synthetic polymers are applied in the treatment of osteonecrosis.

### 4.1 Poly-lactic acid

Lactic acid monomers are used to create PLA, a biodegradable polymer. Medical devices and tissue engineering rely on it for a variety of purposes, including the treatment of osteonecrosis, thanks to its structural adaptability, which allows for the modification of its biodegradation rate and mechanical characteristics. Composite scaffolds, which include PLA among other materials, are commonly used to improve osteogenesis.

For instance, Oliveira et al. (2021) developed scaffolds by incorporating PLA with carbon nanotubes, graphene nanoribbons (GNR), and nano-hydroxyapatite (nHA), producing variations such as nHA/PLA, PLA/GNR, and PLA/nHA/GNR. The PLA/nHA/GNR (3%) scaffold demonstrated the best bone regeneration results, but low GNR concentrations (30 μg/mL) were required to avoid cytotoxicity. Guo et al. (2024) incorporated Mg(OH)_2_ nanoparticles into a PLA scaffold using 3D printing, significantly enhancing the mechanical properties, degradation rate, and bio-mineralization. This PLA/Mg(OH)_2_ scaffold showed long-term magnesium ion release, promoting osteogenesis. Similarly, Wang et al. (2024b) added β-TCP to PLA, creating a PLA/β-TCP scaffold through 3D printing. This scaffold improved compressive strength, bio-mineralization, and biocompatibility, with largest strength of 52.1 MPa in 10% β-TCP content. In the treatment of osteonecrosis, PLA can also serve as a carrier for biomaterials. Hao et al. (Bahraminasab et al., 2022) developed an adenovirus vector carrying human bone morphogenetic protein 2 (hBMP2) and incorporated it into a nano-hydroxyapatite/recombinant human-like collagen/PLA scaffold (nHA/RHLC/PLA). This composite scaffold significantly promoted bone regeneration in rabbit models. Likewise, Maia-Pinto et al. (2021) used PLA and calcium phosphate to create a biological scaffold loaded with hBMP2, enhancing bone repair and osteoinductivity. Jia et al. (2023) combined PLA with nHA and human acellular amnion (HAAM), producing a scaffold that promoted osteoblast adhesion, proliferation, and differentiation. Bahraminasab et al. (2022) created a PLA/G-nHA scaffold, coated by platelet-rich plasma (PRP), which significantly enhanced bone remodeling on a rat osteonecrosis model.

PLA also has been used as drug delivery system for treating osteonecrosis. Gao et al. (2023) developed a PLA/nHA scaffold loaded vancomycin-based chitosan (CS) hydrogel, offering prolonged antibiotic release and enhanced mechanical properties. Li et al. (2023b) similarly created a VAN/PLGA-PLA/nHA scaffold that effectively inhibited *Staphylococcus aureus* growth and improved bone defect repair. Additionally, Liu et al. (2022) designed a PLA/GO scaffold loaded with salvianolic acid B (Sal-B) and aspirin (ASA), which demonstrated enhanced hydrophilicity, cell adhesion, and controlled drug release, supporting long-term bone repair.

In summary, PLA-based scaffolds, when combined with various biomaterials and drugs, show significant potential for osteonecrosis treatment, offering potential solutions for bone engineering and regenerative method.

### 4.2 Polycaprolactone

PCL is a synthetic polymer with excellent biocompatibility and biodegradability, along with desirable softness and plasticity, making it highly suitable for developing intricate biomaterials. Although PCL lacks inherent osteogenic properties, its strong mechanical performance makes it valuable for combining with osteogenic biomaterials in treating osteonecrosis.

**FIGURE 4:**
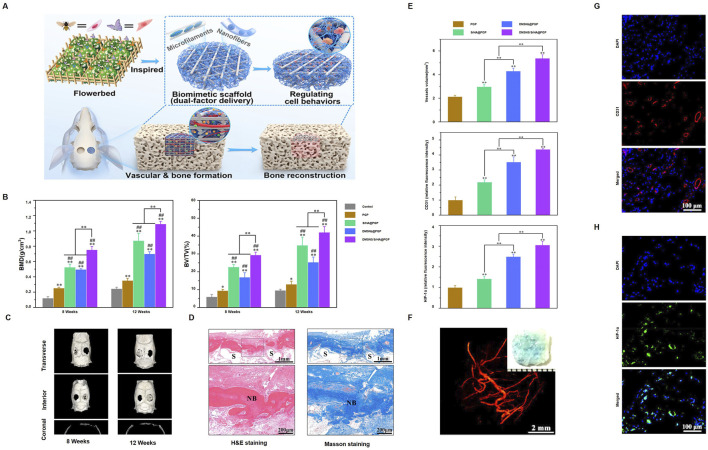
DMSNs/SrHA@PGP-enhanced bone healing: **(A)** Scaffold made by 3D printing and electrospinning, delivering angiogenic and osteogenic agents; **(B)** Micro-CT for bone density and BV/TV (**p < 0.01, ##p < 0.01, *p < 0.05); **(C)** Micro-CT scans at 8 and 12 weeks; **(D)** H&E and Masson staining at 12 weeks (F: fibrous, NB: new bone, S: scaffold); **(E)** Vessel volume and fluorescence; **(F)** Micro-CT of vasculature at 4 weeks; **(G)** CD31 and **(H)** HIF-1α immunofluorescence. (Reproduced with permission [(Zhang et al., 2014)], Copyright 2023, ACS).

PCL is often combined with various materials, including organic and inorganic compounds, metal particles, and biological molecules, to create composite materials for osteonecrosis treatment. Zhou et al. (2023) developed a biomimetic scaffold inspired by a flowerbed design for dual-factor delivery, aimed at promoting vascularization and bone formation (Figure 4A). The DMSN/SrHA@PGP group showed significantly higher bone mineral density (BMD) and bone volume/total volume (BV/TV) at both 8 and 12 weeks (Figure 4B). Micro-CT images showed progressive bone repair in the treated groups (Figure 4C). Histological staining revealed new bone formation in the scaffold area at 8 and 12 weeks (Figure 4D). The DMSN/SrHA@PGP group also had the highest vessel volume, CD31, and HIF-1α expression, indicating improved vascularization and angiogenesis (Figure 4E). A 3D rendering confirmed the extensive vascularization within the scaffold (Figure 4F). Immunofluorescence staining further showed CD31-positive endothelial cells (Figure 4G) and HIF-1α expression (Figure 4H). Kawai et al. (2018) developed a functionally graded PCL/β-TCP scaffold using 3D printing, showing superior bone growth and bone marrow formation in necrotic femoral head tissue, providing a new therapeutic strategy for osteonecrosis. Maia-Pinto et al. (2021) designed a PCL/Willemite (Zn-rich nanoparticles) composite scaffold using 3D printing, which significantly improved osteogenic activity and cell compatibility, providing a novel approach for early-stage osteonecrosis treatment. Saito et al. (2015) created a PCL scaffold loaded with human gingival fibroblasts (HGF) and BMP-7, which significantly enhanced bone growth in mice. Zhang et al. (2014) developed a PCL scaffold coated with indigo-crosslinked GL and rhBMP-2, which promoted osteogenesis and improved bone regeneration *in vitro* and in a nude mouse model.

**FIGURE 5:**
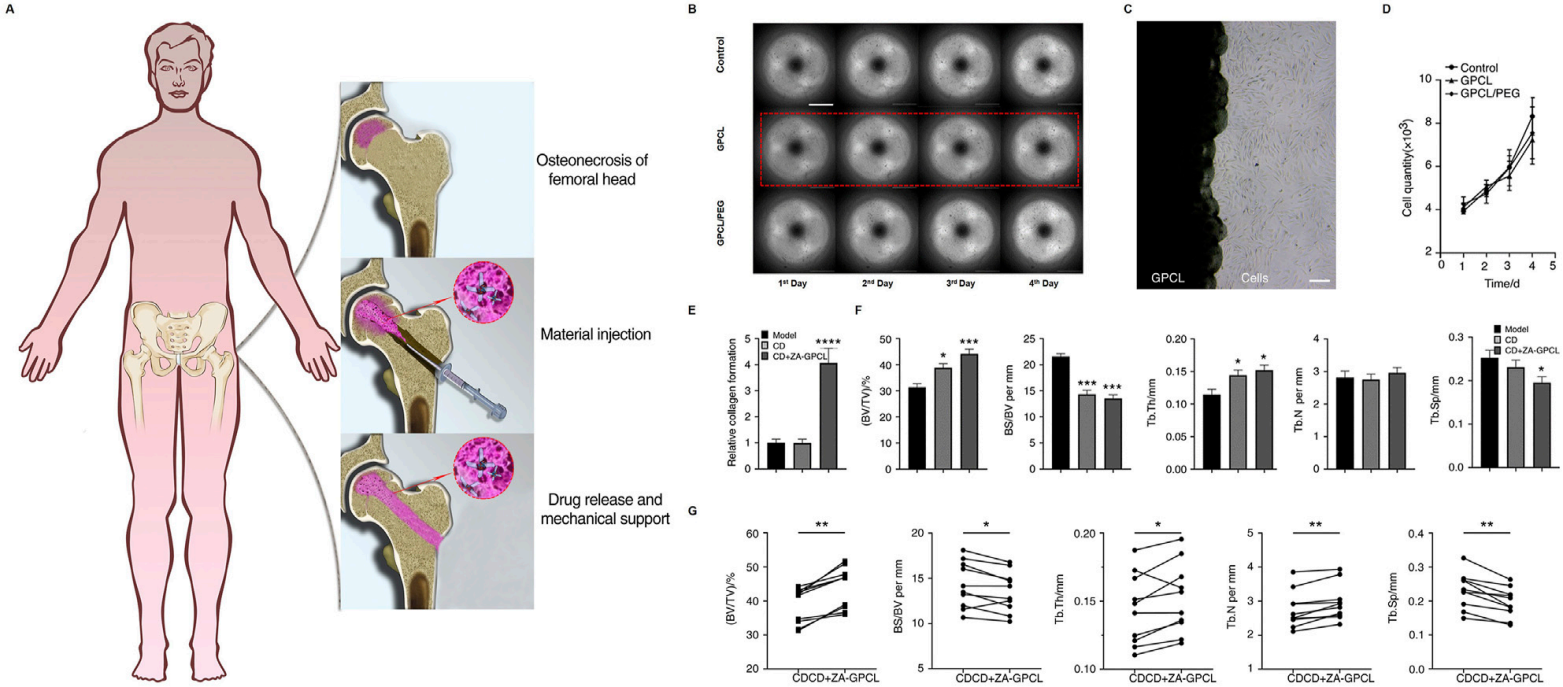
CD + ZA-GPC facilitates bone regeneration: **(A)** Injection methodology; **(B)** MC3T3-E1 cell proliferation in CD + ZA-GPCL extracts; **(C)** Cells cultured with GPCL; **(D)** Proliferation quantification; **(E)** Collagen; **(F)** BV/TV, BS/BV, Tb. N, Tb.Th, Tb. Sp in femoral heads; **(G)** Paired analysis in rabbit femoral heads (*p < 0.05, **p < 0.01, ***p < 0.001). Reproduced with permission [(Nakayama et al., 2017)]; Copyright 2022, Springer Nature.

PCL can also serve as a supportive treatment in combination with other therapies. Kong et al. (2022) demonstrated the use of glycerin-modified polycaprolactone (GPCL) combined with zoledronic acid (ZA) for the treatment of osteonecrosis of the femoral head (ONFH) through core decompression (CD). A schematic illustrates the process, where GPCL is injected into the decompressed bone cavity, serving as a means of drug delivery while providing mechanical support to aid in bone regeneration (Figure 5A). The drug release profiles indicate that GPCL facilitates sustained release over a period of 4 days (Figure 5B). Microscopy results show that GPCL supports excellent cell adherence and growth, and a growth curve further confirms that GPCL promotes higher cell proliferation compared to controls (Figures 5C, D). The CD + ZA-GPCL group exhibited a significantly higher relative collagen formation when it came to bone repair (Figure 5E). The CD + ZA-GPCL group also showed significant improvements in additional metrics related to bone regeneration, such as BV/TV, BS/BV, Tb.Th, Tb.N, and Tb. Sp (Figure 5F). Last but not least, specific data points show that the CD + ZA-GPCL group had better bone density and structural integrity, lending credence to these findings (Figure 5G). According to Zhu et al. (2020) treated steroid-induced osteonecrosis in rats with a composite scaffold containing poly-L-lactic acid, poly-lactic-co-glycolic acid, and polycarbonate (PLLA/PLGA/PCL), bone matrix protein-2 (BMP-2), and low-intensity pulsed ultrasound (LIPUS). This scaffold improved bone formation, angiogenesis, and differentiation. Research like this shows how flexible PCL-based materials can be for treating osteonecrosis and encouraging bone regeneration, particularly when mixed with other materials and therapies.

### 4.3 Poly-lactic-co-glycolic acid

PLGA is a biodegradable copolymer composed of polylactic acid (PLA) and polyglycolic acid (PGA). It integrates the advantageous characteristics of both components, providing exceptional biocompatibility and degradability, rendering it an appropriate material for medical applications, especially in tissue engineering and drug delivery systems. Modifying the PLA-to-PGA ratio allows for the customization of PLGA’s physicochemical properties to fulfill particular requirements. PLGA has been utilized in multiple methods to address osteonecrosis (Yoon and Chung, 2022; Nakayama et al., 2017).

**FIGURE 6:**
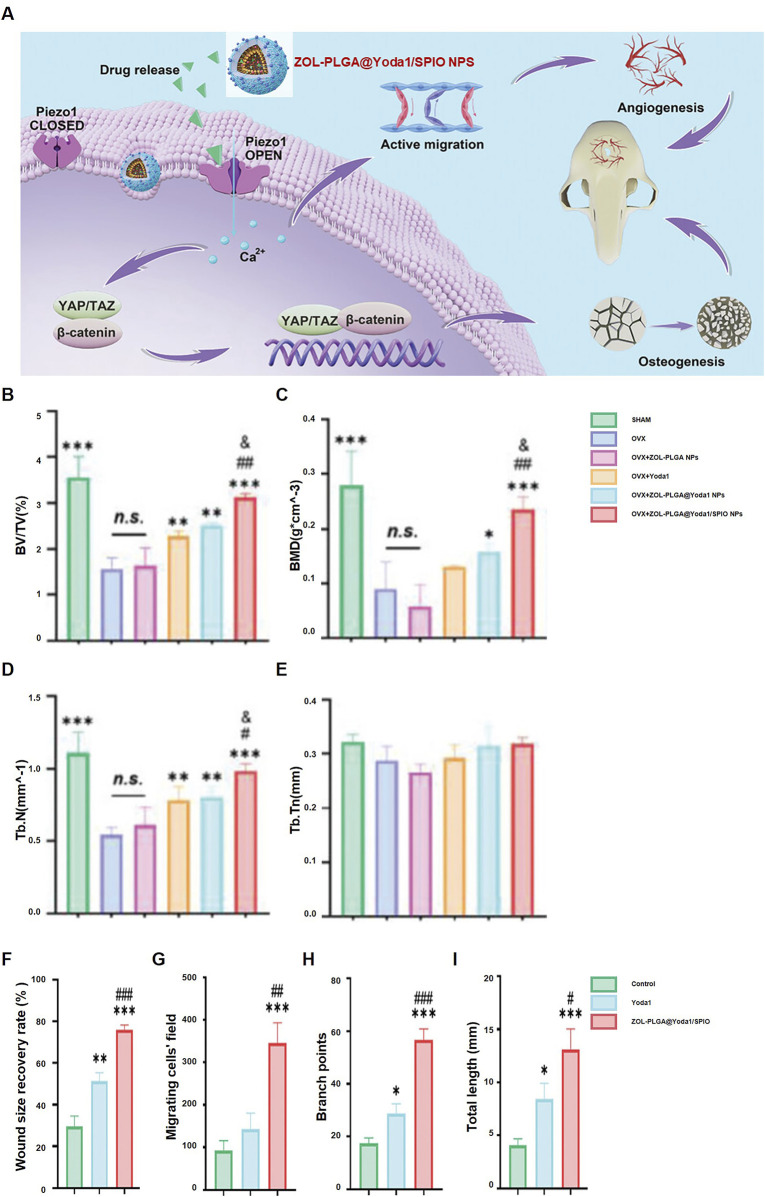
ZOL-PLGA@Yoda1/SPIO nanoparticles facilitate bone regeneration: **(A)** Mechanism of therapy for osteoporotic defects; **(B–E)** Micro-CT quantification: BV/TV **(B)**, BMD **(C)**, Tb.N **(D)**, Tb.Th **(E)** (*p < 0.05, **p < 0.01, ***p < 0.001 compared to OVX); **(F–I)** Wound healing **(F)**, cell migration **(G)**, branch points **(H)**, tube length **(I)** (*p < 0.05, **p < 0.01, ***p < 0.001 compared to Control, #p < 0.05, ##p < 0.01 compared to Yoda1). Reproduced with permission [(Gu et al., 2024)], Copyright 2023, Wiley-VCH GmbH.

A promising application of PLGA is in nanomaterials for osteonecrosis treatment. Guan et al. (2024) dFormulated magnetic PLGA nanoparticles (ZOL-PLGA@Yoda1/SPIO NPs) that enhance osteogenesis and angiogenesis. These nanoparticles promote bone regeneration and vascular remodeling by activating Piezo1 channels, resulting in calcium influx and the subsequent activation of the YAP/TAZ and β-catenin pathways (Figure 6A). The ZOL-PLGA@Yoda1/SPIO NPs group markedly enhanced bone volume/total volume (BV/TV) (Figure 6B), bone mineral density (BMD) (Figure 6C), trabecular number (Tb.N) (Figure 6D), and trabecular thickness (Tb.Th) (Figure 6E). The wound healing rate and cellular migration were significantly improved in this cohort (Figures 6F, G). Angiogenesis metrics, including branch points and total vessel length, were also significantly improved (Figures 6H, I). Hassan et al. (2024) optimized PLGA nanoparticles for improved biocompatibility and drug release, significantly enhancing osteonecrosis treatment. Zhou et al. (2024) developed a 3D-printed PLGA/β-TCP/Mg scaffold, which promoted bone regeneration and improved mechanical strength for cranial osteonecrosis. Gu et al. (2024) created a composite scaffold with Ti3C2Tx@PLGA/icariin/β-TCP, where icariin release, regulated by near-infrared response, promoted bone regeneration. Huang et al. (2024) designed an injectable bone cement composed of PLGA, CPC, ALN, and MgO, which inhibited osteoclast activity and promoted osteogenesis in osteonecrotic areas.

PLGA also contributes significantly in cell therapy. Kessler et al. (2024) used PLGA scaffolds loaded with mouse iPSCs to enhance regeneration in large osteonecrotic areas, providing new avenues for bone regeneration therapy. Rodrigues et al. (2024) manufactured bioresorbable PLGA composite membranes that demonstrated good osteoconductivity and biocompatibility, offering potential for osteonecrosis treatment.

Furthermore, PLGA serves as an effective drug carrier. Fan et al. (2024) developed a PLGA-based scaffold containing pioglitazone-loaded nanospheres, which modulated the immune microenvironment and promoted angiogenesis at osteonecrotic sites. Annaji et al. (2024) designed a 3D-printed PLGA implant scaffold with gradual drug release, enhancing bone regeneration in osteonecrotic areas.

Research has demonstrated the efficacy of combining PLGA with PCL for the treatment of osteonecrosis. Qian et al. (2019) Engineered a silver-modified, collagen-coated electrospun PLGA/PCL scaffold that enhanced antibacterial properties and osteogenic efficacy. Zhou et al. (2018) developed a 3D-printed PCL scaffold infused with PLGA microspheres encapsulating vancomycin, ensuring prolonged antibacterial efficacy. Qian et al. (2016) created PLGA/PCL membranes that significantly promoted osteoblast attachment and growth. Wang et al. (2019) demonstrated that adding octacalcium phosphate (OCP) to PLGA/PCL nanofiber membranes improved osteoinductive abilities and mechanical properties. Won et al. (2016) developed a 3D-printed PCL/PLGA/β-TCP membrane that demonstrated strong bone growth effects and enhanced mechanical properties with wet environments.

In conclusion, PLGA has proven to be a versatile material for osteonecrosis treatment, whether used alone or in combination with other materials like PCL, showcasing considerable promise in osseous tissue engineering and drug transport platform.

## 5 Bone marrow mesenchymal stem cells

Promoting bone regeneration is an essential part of treating osteonecrosis. Angiogenesis, matrix production, cell proliferation, and differentiation are all components of bone regeneration. The pluripotency and self-renewal capacities of stem cells, especially BMMSCs, are crucial to this process. Because of their high osteogenic capacity and accessibility, BMMSCs see extensive utilization.

BMMSCs enhance osteogenesis through gene expression regulation. Yang et al. (2023) found that microRNA Let-7a upregulates the Fas/FasL signaling pathway, enhancing autophagy in BMMSCs and improving their osteogenic differentiation capacity, promoting bone formation in osteonecrosis. Ohori-Morita et al. (2022) used neurosphere culture techniques to maintain stem cell characteristics in BMMSCs, ensuring effective osteoblast differentiation after transplantation into a bone defect model.

BMMSCs also regulate the microenvironment to promote osteogenesis. Baron et al. (2023) demonstrated that BMMSCs reduced local inflammation by regulating macrophages and T cells, creating a favorable environment for bone regeneration. Campos Totoli et al. (2023) demonstrated that the sequential injection of BMMSCs alongside adipose-derived stem cells into a rat cranial defect model enhanced the tissue microenvironment, enhancing the osteogenic effect of the combined stem cells. Mastrolia et al. (2022) found that BMMSCs directly promote new bone formation, regulate the necrotic microenvironment, inhibit inflammation, and enhance bone tissue repair. BMMSCs also promote angiogenesis. Deng et al. (2024) demonstrated that BMMSCs increase vascular endothelial growth factor (VEGFA) expression, promoting endothelial cell migration and angiogenesis in osteonecrosis. Hua et al. (2024) discovered that nuclear fibrous membranes improve BMMSC osteogenic differentiation by activating the mitochondrial SIRT3 pathway and promoting vascularized bone repair.

BMMSCs can enhance osteonecrosis treatment when combined with nanomaterials. Zhang et al. (2024) showed that zinc oxide nanowires release zinc ions, which improve BMMSC adhesion, proliferation, and osteogenic differentiation. Lv et al. (2024) demonstrated that melatonin-supported nanofiber scaffolds improve BMMSC mitochondrial function, promoting bone matrix deposition and vascularization.

BMMSCs also show synergy with other cells in osteogenesis. Sawada et al. (2023) showed that co-injection of BMMSCs and BM-DFATs enhanced osteogenic capacity and accelerated bone regeneration. Baek et al. (2022) reported that co-culturing growth plate cells (EGPCs) with BMMSCs significantly increased chondrogenic and osteogenic differentiation markers, improving bone regeneration in osteonecrotic areas, especially in osteoporosis models.

## 6 Discussion and outlook

The diagnosis and medical management of osteonecrosis remain challenging in clinical practice due to its intricate pathological progression and multifactorial origins. Biomaterials possess significant potential for enhancing bone regeneration, offering new avenues for treating osteonecrosis-related conditions when prosthetic replacement is not a therapeutic option. This review offers a comprehensive look at biomaterials, including bioceramics and both natural and synthetic polymers, and stem cell-based approaches, which show potential for improving osteogenesis and angiogenesis. These materials can alter the bone microenvironment, affect cellular functions, and transport bioactive agents to facilitate bone repair. Nonetheless, numerous challenges related to biomaterials must still be addressed before they can be considered standard clinical treatments for osteonecrosis.

First of all, the mechanical properties of biomaterials have to be optimized, mainly for the load-bearing bones. While natural polymers and bioceramics offer excellent biocompatibility and bioactivity, they often fall short in providing the mechanical strength required for supporting high-stress areas. This is what future research on composite biomaterials should address: their adequate mechanical properties would be combined with osteogenic properties, thus allowing these materials to support the process of regeneration without loss of structural integrity.

A very important issue is biomaterial degradation in a controlled manner. The degradation rate must align with the formation of new bone to ensure the material provides adequate support throughout the healing process. If this material degrades too quickly, too little bone may form; if it degrades too slowly, it interferes with natural repair processes. Biomaterials with controllable and predictable degradation rates hold the future for optimization in treating osteonecrosis.

Increased bioactivity was another direction to be developed in the future. Incorporating bioactive ions, growth factors, or other active molecules would significantly improve bioactivity and bone regeneration performance. Trace element doping ventured into using lithium and copper ions, which further improved both bone regeneration and vascular remodeling. Key ongoing research has worked on incorporating potential bioactive agents into biomaterials, thus hastening the healing process in the osteonecrosis therapies more efficiently.

The trend in immunomodulation does seem very promising for this area of research. Indeed, different recent studies have showed the drastic amplification that biomaterials with immune-modulating capabilities-more importantly, those shifting macrophages toward a healing-promoting phenotype-exert on tissue regeneration. In the future, biomaterials for osteonecrosis will arguably be more functional in immune modulation and inflammation reduction, and in creating an environment much more suitable for bone repair.

Personalized therapies and translation into the clinic are the final essential aspects that are to be envisaged from research. The condition varies individually in cause, extent of necrosis, and general health; thus, personalized biomaterials stand as a promising solution in this regard. Advances in 3D printing and biofabrication may, in the future, provide the creation of scaffolds customized to meet the specific needs of each patient. Overcoming major regulatory, manufacturing, challenges will allow the implementation of such advanced materials clinically. Currently, some studies have validated the efficacy and safety of 3D printed personalized materials, but the overall progress of translating these materials into clinical trials remains slow (Dong et al., 2020; Long et al., 2021; Luo et al., 2023; Bai et al., 2025). The main reasons include challenges in technology, production, and regulation. While 3D printing has achieved preliminary success in laboratory and animal models, ensuring the long-term stability, immune response, and compatibility with surrounding tissues in human applications remains an issue to be addressed. Furthermore, the scaling up of production, cost control, and the standardization of production processes that meet clinical needs are not yet fully developed, which limits its widespread application.

To accelerate the translation process, more interdisciplinary collaboration is required, particularly between materials science, clinical medicine, and bioengineering. Conducting large-scale, multi-center clinical trials, especially verifying the effectiveness of personalized treatments in different pathological contexts, will provide stronger evidence for the clinical application of 3D printed personalized materials. At the same time, regulatory agencies should strengthen cooperation with researchers and industry to establish more forward-looking policies and regulations to facilitate the rapid and safe clinical application of new technologies.
